# Supplementary material for: Genetic Diversity and Characterization of Circular Replication (Rep)-Encoding Single-Stranded (CRESS) DNA Viruses
Source: Microbiol Spectr. 2022 Nov 8;10(6):e01057-22. doi: 10.1128/spectrum.01057-22 (PMC9769708; doi:10.1128/spectrum.01057-22)
Supplement: Supplemental file 10 — Fig. S1 to S5. Download spectrum.01057-22-s0010.pdf, PDF file, 34.4 MB [file spectrum.01057-22-s0010.pdf]

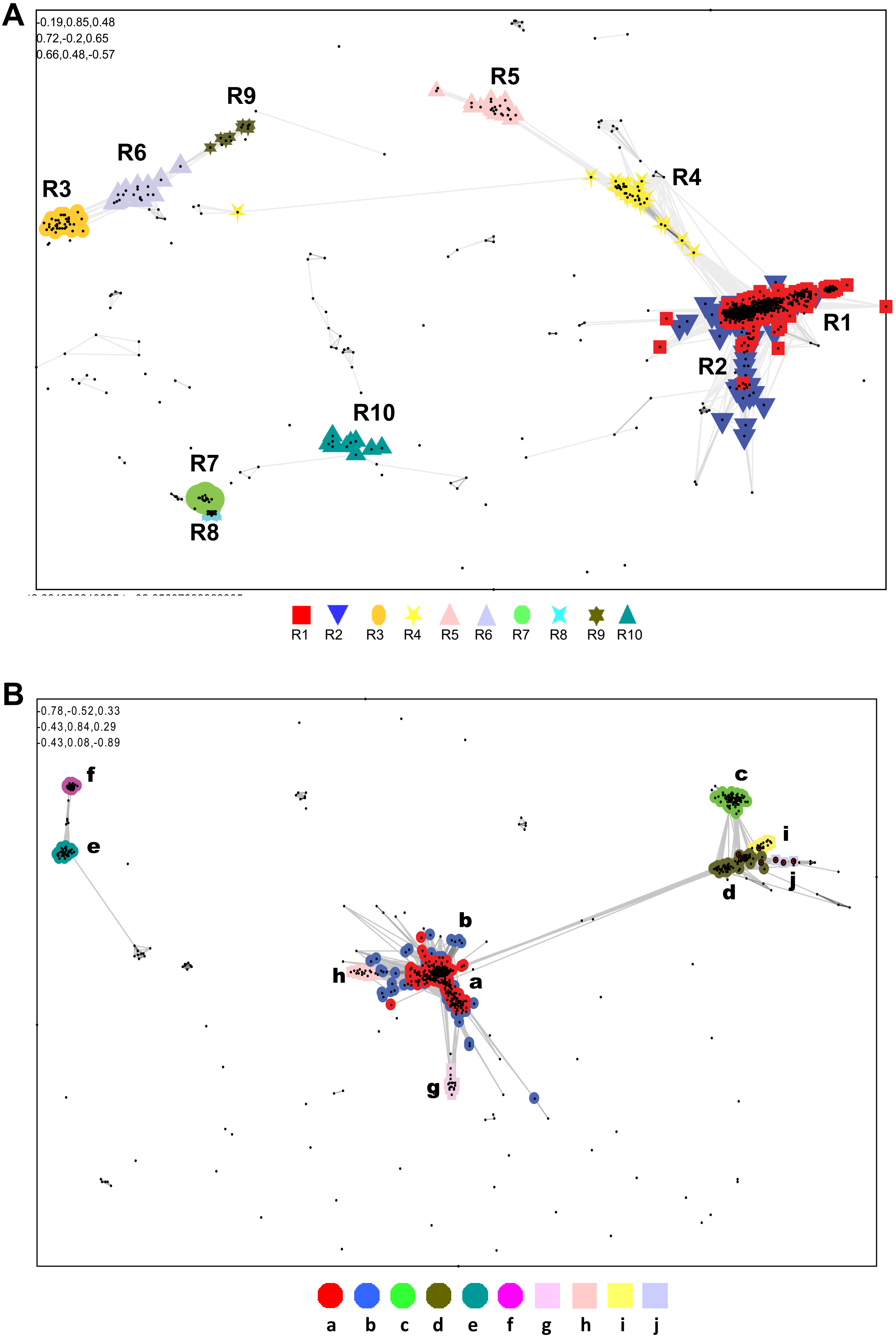

Supplementary Figure 1

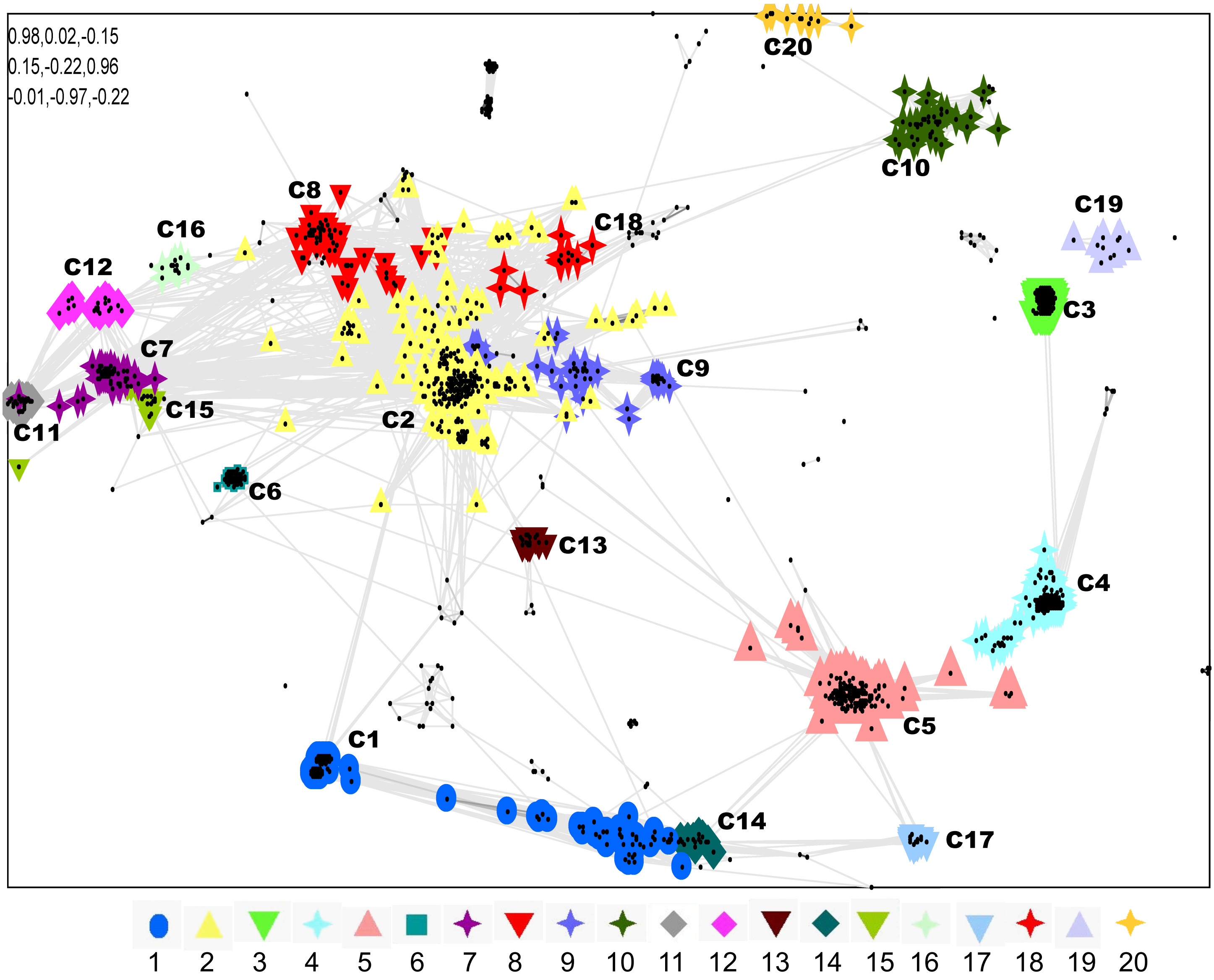

Supplementary Figure 2

A

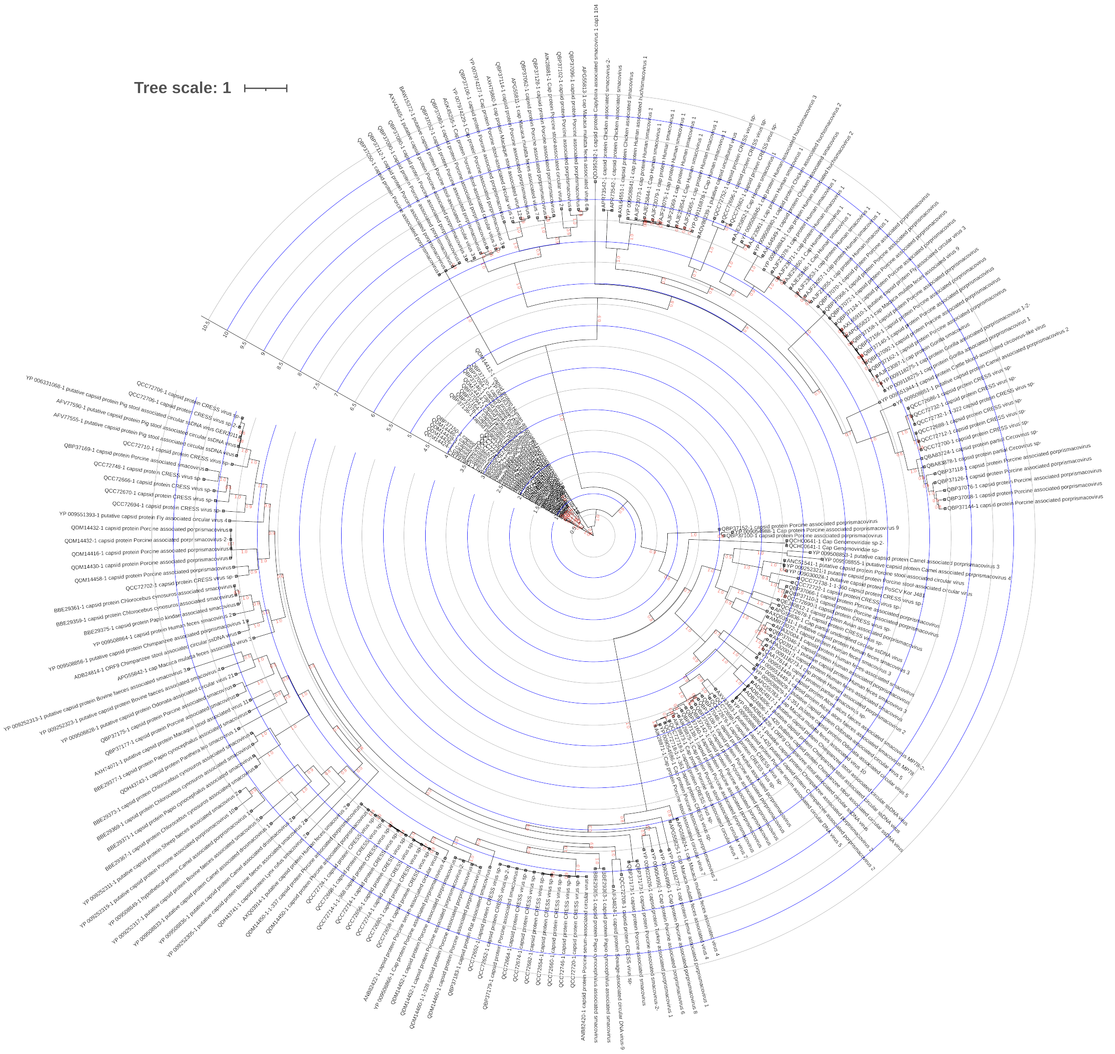

B

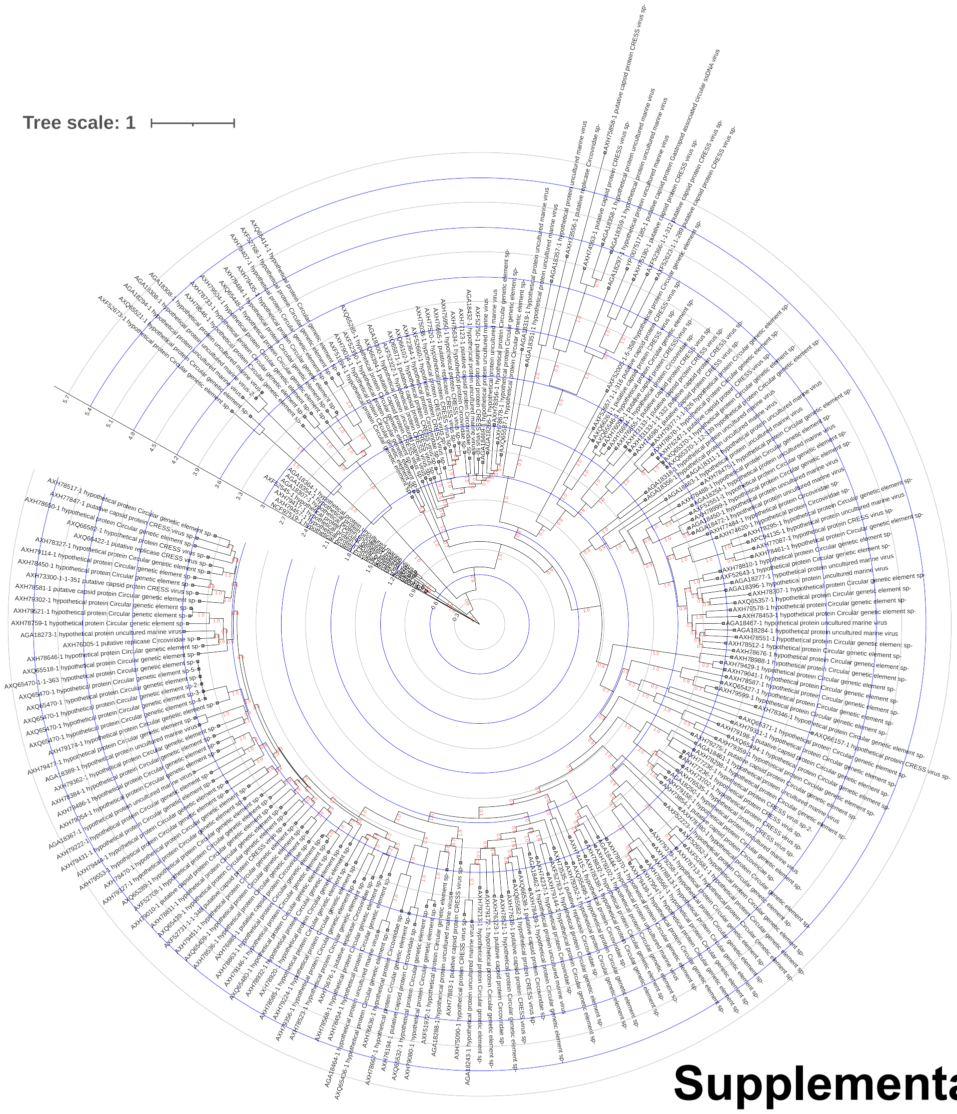

Supplementary Figure 3

A

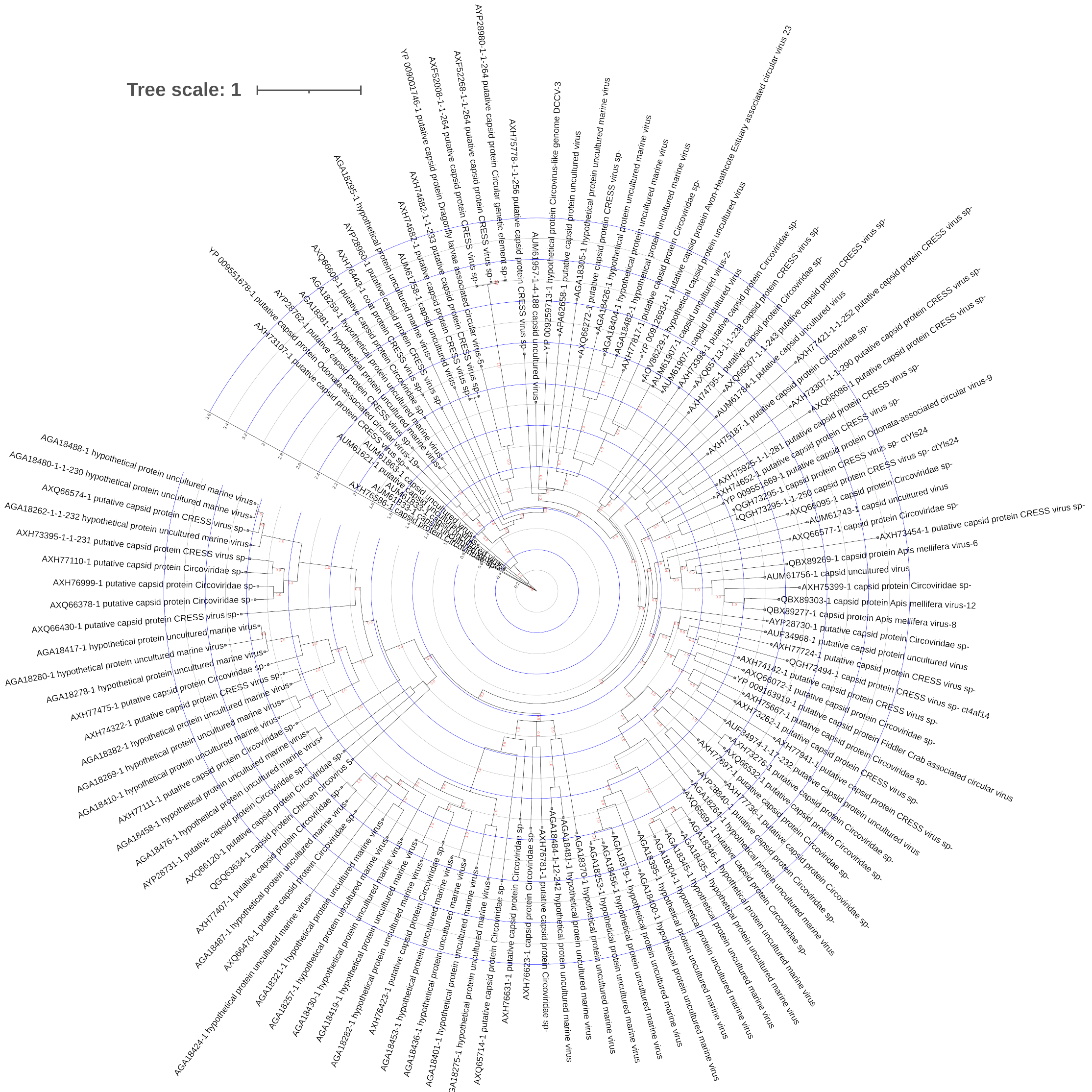

B

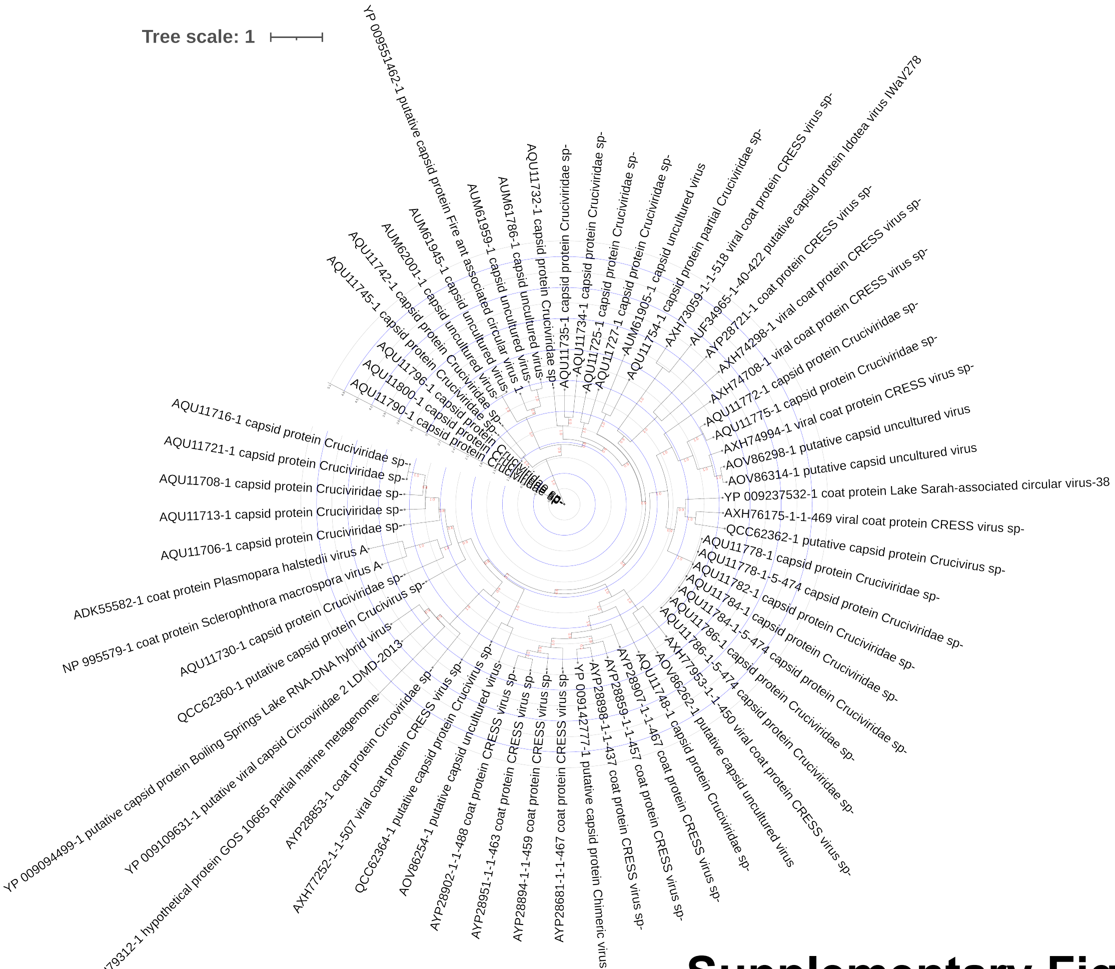

Supplementary Figure 4

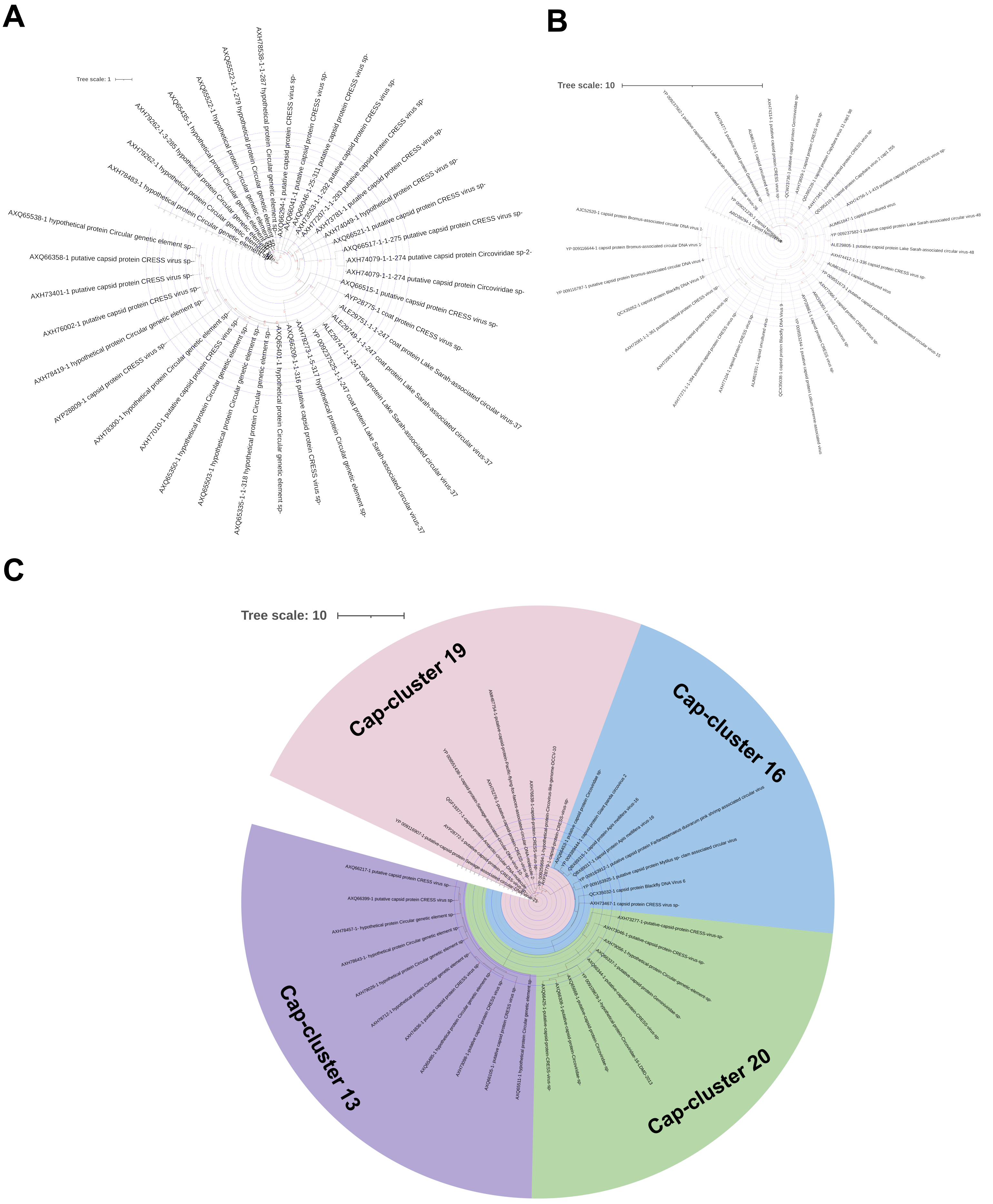

Supplementary Figure 5

Supplementary Figure Legends

Supplementary Figure 1: Pairwise amino acid sequence similarity network-based CRESS-DNA virus classification of Rep protein of CRESS-DNA viruses. (**A**) A total of 1160 amino acid sequences of Rep protein of CRESS-DNA viruses (**Supplementary Data 1**) were clustered by CLANS. The clusters were classified using the Network-based method using offset values and global average with a maximum of 10000 in CLANS Toolkit analysis. The *P*-value $\leq 1e^{-05}$ was used to show the lines connecting the sequences. (**B**) Sub-clustering of the cluster 1 and 2 protein sequences of CRESS-DNA viruses Rep proteins into 10 different clusters (cluster **a** to **j**) at a *P*-value threshold of $1e^{-38}$.

Supplementary Figure 2: A total of 1823 amino acid sequences of Cap protein of CRESS-DNA viruses were (**Supplementary Data 6**) used and classified by their pairwise sequence similarity network using CLANS. The clusters were classified using the Network-based method using offset values and global average with a maximum of 10000 in CLANS Toolkit analysis. The *P*-value $\leq 1e^{-02}$ was used to show the lines connecting the sequences.

Supplementary Figure 3: Phylogenetic relationship of CRESS-DNA virus Cap protein cluster C3 and C4. (**A**) Phylogenetic tree depicting the genetic relationship between the CRESS-DNA virus Cap protein cluster C3. The details of sequences in the cluster (**Supplementary Data 7**) and alignment are provided in **Supplementary Data 12**. (**B**) Phylogenetic tree depicting the genetic relationship between the CRESS-DNA virus Cap protein cluster C4. The details of sequences in the cluster (**Supplementary Data 7**) and alignment are provided in **Supplementary Data 13**. The maximum-likelihood method inferred the evolutionary history using the Subtree-Pruning-Regrafting algorithm and bootstrap values in PhyML 3.3_1.

Supplementary Figure 4: Phylogenetic relationship of CRESS-DNA virus Cap protein cluster C6 and C7. (**A**) Phylogenetic tree depicting the genetic relationship between the CRESS-DNA virus Cap protein cluster C6. The details of sequences in the cluster (**Supplementary Data 7**) and alignment are provided in **Supplementary Data 14**. (**B**) Phylogenetic tree depicting the genetic relationship between the CRESS-DNA virus Cap protein cluster C7. The details of sequences in the cluster (**Supplementary Data 7**) and alignment are provided in **Supplementary Data 15**. The maximum-likelihood method inferred the evolutionary history using the Subtree-Pruning-Regrafting algorithm and bootstrap values in PhyML 3.3_1.

Supplementary Figure 5: Phylogenetic relationship of CRESS-DNA virus Cap protein cluster C10, C11 C13, C16, C19, and C20. (**A**) Phylogenetic tree depicting the genetic relationship between the CRESS-DNA virus Cap protein cluster C10. The details of sequences in the cluster (**Supplementary Data 7**) and alignment are provided in **Supplementary Data 16**. (**B**) Phylogenetic tree depicting the genetic relationship between the CRESS-DNA virus Cap protein cluster C11. The details of sequences in the cluster (**Supplementary Data 7**) and alignment are provided in **Supplementary Data 17**. (**B**) Phylogenetic tree depicting the genetic relationship between the CRESS-DNA virus Cap

protein cluster C13, C16, C19, and C20. The details of sequences in each cluster (**Supplementary Data 7**) and alignment are provided in **Supplementary Data 18**. The maximum-likelihood method inferred the evolutionary history using the Subtree-Pruning-Regrafting algorithm and bootstrap values in PhyML 3.3_1.
